# Exploratory immunomonitoring during radiochemotherapy in HNSCC and machine-learning reveal immune parameters associated with disease-free survival

**DOI:** 10.1038/s41698-026-01658-w

**Published:** 2026-08-31

**Authors:** Anna-Jasmina Donaubauer, Oliver Tomic, Lia Mogge, Sarina K. Müller, Cecilia Marie Futsaether, Kristian Hovde Liland, Bao Ngoc Huynh, Jens von der Grün, Panagiotis Balermpas, Max Fleischmann, Matthias G. Hautmann, Felix Steger, Christopher Bohr, Thomas Hehr, Carmen Stromberger, Volker Budach, Markus Schymalla, Rita Engenhart-Cabillic, Lukas Kocik, Hans Geinitz, Ursula Nestle, Gunter Klautke, Claudia Scherl, Philipp Schubert, Stefanie Corradini, Rainer Fietkau, Udo S. Gaipl, Benjamin Frey, Marlen Haderlein

**Affiliations:** 1 Translational Radiobiology, Department of Radiation Oncology, Universitätsklinikum Erlangen, Friedrich-Alexander-Universität Erlangen-Nürnberg, Erlangen, Germany; 2 Department of Radiation Oncology, Universitätsklinikum Erlangen, Friedrich-Alexander-Universität Erlangen-Nürnberg, Erlangen, Germany; 3 Comprehensive Cancer Center Erlangen-EMN, Erlangen, Germany; 4 Bavarian Cancer Research Center (BZKF), Erlangen, Germany; 5 Norwegian University of Life Sciences, Faculty of Science and Technology, Ås, Norway; 6 Department of Otolaryngology - Head & Neck Surgery, Universitätsklinikum Erlangen, Friedrich-Alexander-Universität Erlangen-Nürnberg, Erlangen, Germany; 7 Department of Medical Physics, Oslo University Hospital, Oslo, Norway; 8 Department of Radiation Oncology, Zurich University Hospital, Zurich, Switzerland; 9 Department of Radiotherapy and Radiation Oncology, University Hospital Frankfurt, Goethe-Universität Frankfurt am Main, Frankfurt am Main, Germany; 10 Department of Radiotherapy and Radiation Oncology, University Hospital of Regensburg, Regensburg, Germany; 11 Department of Otorhinolaryngology, University Hospital Regensburg, Regensburg, Germany; 12 Department of Radiotherapy and Radiation Oncology, Marienhospital, Stuttgart, Germany; 13 Department of Radiotherapy and Radiation Oncology, Charité - Universitätsmedizin Berlin, Corporate Member of Freie Universität Berlin and Humboldt-Universität zu Berlin, Berlin, Germany; 14 Department of Radiotherapy and Radiation Oncology, University Hospital of Marburg, Marburg, Germany; 15 Department of Radiation Oncology and Radiotherapy, Ordensklinikum Linz Barmherzige Schwestern, Linz, Austria; 16 Faculty of Medicine, Johannes Kepler University Linz, Linz, Austria; 17 Department of Radiotherapy and Radiation Oncology, Kliniken Maria Hilf, Moenchengladbach, Germany; 18 Department of Radiation Oncology, Chemnitz Hospital, Chemnitz, Germany; 19 Department of Otorhinolaryngology, Head and Neck Surgery, University Hospital Mannheim, Medical Faculty Mannheim, Heidelberg University, Mannheim, Germany

**Keywords:** Biomarkers, Cancer, Computational biology and bioinformatics, Immunology, Oncology

## Abstract

Immunological biomarkers are increasingly relevant for personalized cancer treatment, but peripheral blood-derived biomarkers are not yet used to guide therapy in head and neck squamous cell carcinoma (HNSCC). The prospective non-randomized DIREKHT study (ClinicalTrials.gov: NCT02528955, 2015-08-19) therefore integrated immune monitoring into postoperative radio(chemo)therapy (R(C)T) to explore blood-based biomarkers. In 70 oral cavity and oropharyngeal cancer patients receiving curative R(C)T, the peripheral immune status was assessed before and after therapy and during follow-up by flow cytometry-based immunophenotyping of 45 immune parameters. A machine learning workflow identified predictors of disease-free survival (DFS), using Repeated Elastic Net Technique (RENT) feature selection within repeated stratified K-fold cross-validation and nested cross-validation for tuning and assessment. This approach identified a 29-parameter immune signature from pre- and post-therapeutic profiles, with key contributors including HLA-DR + T cells, HLA-DR+ monocytes, and basophils. The best model achieved a Matthews correlation coefficient of 0.681, with pre- and post-therapeutic parameters contributing equally, highlighting immune dynamics during R(C)T. Adding clinical parameters did not improve performance (MCC = 0.678), but yielded a comparable model integrating immune and clinical variables. Blood-based immune signatures may have prognostic relevance for DFS after R(C)T in HNSCC. Validation in larger cohorts is required to confirm clinical applicability and reduce the signature.

## Introduction

Tumors arising in the mucosal tissues of the head and neck region, such as the oral cavity or the pharynx and larynx are referred to as head-and-neck squamous cell carcinomas (HNSCC). Around 900,000 cases worldwide are identified per year, and in Germany, around 50 out of 100,000 people are diagnosed with HNSCC every year^1,2^. The main drivers of this cancer are lifestyle factors such as the consumption of alcohol and tobacco or infections with oncogenic strains of the human papillomavirus (HPV)^3^. The global incidence of HNSCC is expected to rise by around 30% in 2030. This trend is driven mainly by increasing HPV-related HNSCC cases and persistent alcohol and tobacco consumption in developing countries. In industrialized nations such as Germany and the United States, the HPV-related cases are also rising, whereas the rate of tobacco- and alcohol-related HNSCC is declining^1^. HNSCC is a heterogeneous tumor disease and is often diagnosed at locally advanced disease stages. Thus, treatment is complex and includes various treatment modalities such as surgery, radiation therapy (RT), chemotherapy (CT) and immunotherapy (IT) with immune checkpoint inhibitors (ICIs)^3^.

Immunological biomarkers are becoming increasingly important in personalized treatment approaches for cancer^4,5^. The successful integration of ICIs as a therapeutic option in HNSCC, points to the importance of analyzing the role of the immune system in this cancer entity^6^. In the palliative situation, ICIs are standard of care in HNSCC, whereas in patients undergoing definitive curative radio-(chemo)therapy (R(C)T), most studies so far failed to demonstrate a benefit of adding IT to R(C)T^7,8^. In the perioperative and postoperative situation, several studies showed a clinical benefit of adding ICI to R(C)T in locally advanced HNSCC with programmed death-ligand 1(PD-L1) expression and a combined positive score ≥1%^9,10^ and perioperative Pembrolizumab in resectable HNSCC with a PD-L1 CPS ≥1% is the standard of care. Currently, the only validated and clinically applied immunological biomarker for ICI therapy in HNSCC is the expression of PD-L1 on tumor cells. This marker relies on analyses from tumor biopsies, which are difficult to obtain longitudinally. Also, the PD-L1 expression on tumor cells has other shortcomings that limit its significance as a biomarker, including only a moderate correlation with the outcomes of patients and a dynamic expression of PD-L1 that might be influenced by previous treatments such as RT and CT^11^. Further, the validity of the PD-L1 expression is strongly dependent on the immunological context and thus, other parameters such as T cell infiltration and density or the co-expression of alternative checkpoints like T-cell immunoglobulin and mucin-domain containing-3 (TIM-3) or Lymphocyte-activation gene 3 (LAG-3) should be considered^6,7,12^.

Liquid biopsies that can be obtained from peripheral blood enable the acquisition of a set of immune markers, including various cell types and activation and immune checkpoint markers. They are also available longitudinally throughout the course of therapy, potentially capturing treatment-induced changes in the immune status^13,14^. In addition, therapeutic biomarkers are essential, particularly in immunotherapy regimens (e.g. Keynote 689 trial), to accurately predict potentially serious side effects^9^. In line with that, predictive immune cell populations could be identified in previous analyses of HNSCC patients receiving ICI therapy for locally advanced or metastasized disease states. In these studies, monocytes, cytotoxic T cells, plasmacytoid dendritic cells and granulocytes, especially the neutrophilic granulocytes, predicted the patient outcome^4,15^.

These studies, however, focused only on advanced HNSCC disease stages and patients receiving ICI therapy. Nonetheless, the analysis of the longitudinal immune status of patients who receive R(C)T, also in less advanced disease stages, is of major importance^16^. It is well-known that RT and CT modulate the immune system and the anti-tumoral immune response^17,18^, and that many patients receiving ICIs have previously undergone treatment modalities such as R(C)T^19,20^. Thus, the predictive power of the R(C)T-induced immune modulations needs to be unraveled.

To address these issues and to define immunological biomarkers for HNSCC, the prospective DIREKHT study (NCT02528955) integrated a translational research program, in which the longitudinal immune status of HNSCC patients was monitored throughout their treatment with R(C)T. The aim was to identify predictive immune signatures from the peripheral blood of patients for prognosis and therapy optimization of HNSCC.

## Results

### The best predictive model is built from the longitudinal immunological dataset alone

Before developing predictive models using the ML pipeline, a principal component (PCA) and subsequent Analysis of Variance (ANOVA) were applied to test for systematic differences in the immune status of patients in study arms B and C, either at baseline (T1) or after therapy (T2). Further details on the PCA and ANOVA and the respective results can be found in the supplementary material and Supplementary Fig. 1 & Supplementary Table 1. No differences in the immune status of both patient cohorts were found, justifying combining the cohorts for modeling.

**Fig. 1:**
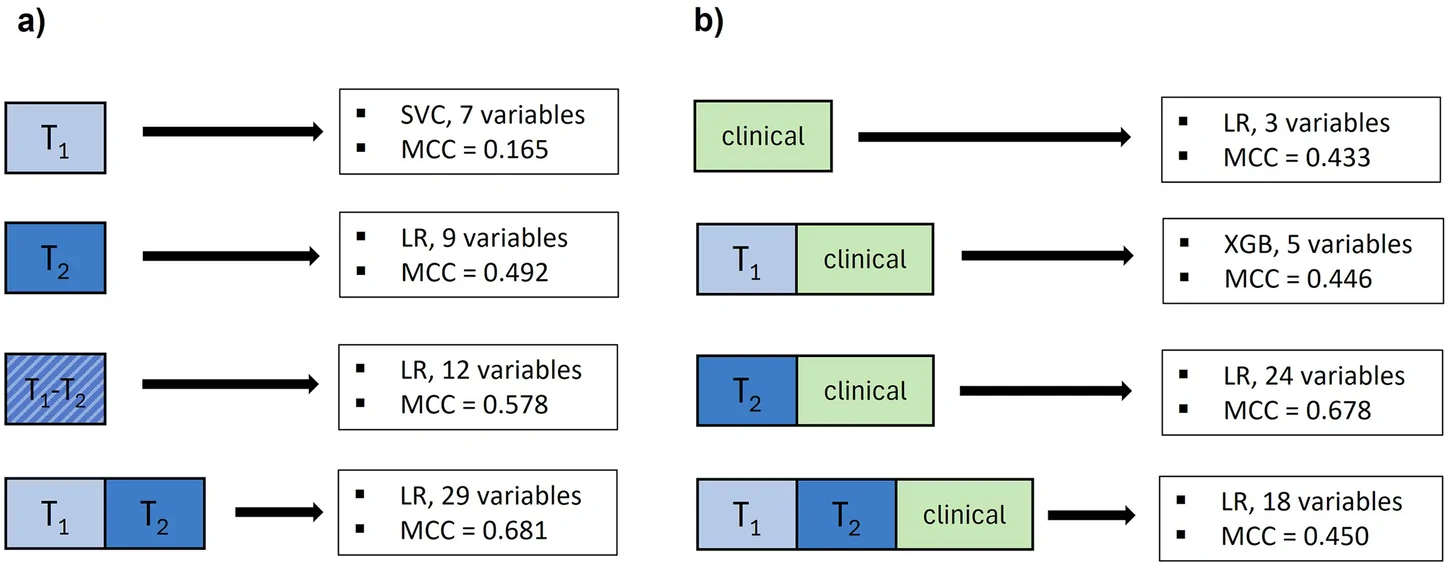
Performance of the predictive models trained on different dataset combinations. Different combinations of the immunological and clinical datasets were used to train machine learning models for the prediction of disease-free survival. Model performance is given by the metrics Matthews correlation coefficient (MCC). T1 denotes the pre-therapeutic immunological dataset, and T2 denotes the post-therapeutic immunological dataset. Light blue rectangles indicate T1 data, dark blue rectangles indicate T2 data, hatched blue rectangles indicate the T1–T2 dataset, and green rectangles indicate clinical data. White rectangles summarize the best-performing machine learning algorithm (support vector machine (SVM), logistic regression (LR), extreme gradient boosting (XGB)), the number of selected variables, and the corresponding MCC value for each dataset combination. In (**a**), the models were trained using immunological datasets only, including T1, T2, T1–T2, and the combined T1 plus T2 dataset. In (**b**), models were trained using clinical data alone or in combination with the immunological datasets T1 and/or T2.

The performance of the predictive models based on the immunological datasets T1 and T2, and the clinical dataset are summarized in Fig. 1 and Table 1. First, the immunological datasets alone were used for training and validation (Fig. 1a). Modeling with the pre-therapeutic immune status alone (${X}_{T1}$) led to a model performance of MCC = 0.165 with SVC. A higher model performance was achieved when the post-therapeutic immune status dataset (${X}_{T2}$) was used. Here, LR resulted in an MCC = 0.492. To consider the dynamics of the immune status during therapy, we next trained the model on the differences between the immune variables from the pre- to the post-therapeutic evaluation (${X}_{{diffT}1T2}$), resulting in a further increase in model performance to an MCC = 0.578. Finally, the complete immunological dataset containing all variables from the pre- and post-therapeutic immune status (${X}_{T1T2}$) achieved the highest performance of MCC = 0.681 using LR.

**Table 1:** Average predictive performance on the test sets (outer folds) from nested cross-validation of the machine learning models based on different datasets

Data	Best algorithm	MCC	Scaled MCC	AUC	F1 pos	F1 neg	Acc	Number of variables	RENT selection frequency
${{\boldsymbol{X}}}_{{\boldsymbol{T}}{\boldsymbol{1}}}$	SVC	0.165 (±0.32)	0.583 (±0.16)	0.599 (±0.19)	0.230 (±0.27)	0.888 (±0.06)	0.810 (±0.09)	7	20
${{\boldsymbol{X}}}_{{\boldsymbol{T}}{\boldsymbol{2}}}$	LR	0.492 (±0.26)	0.746 (±0.13)	0.726 (±0.13)	0.512 (±0.24)	0.943 (±0.03)	0.900 (±0.05)	9	16
${{\boldsymbol{X}}}_{{\boldsymbol{diffT}}{\boldsymbol{1}}{\boldsymbol{T}}{\boldsymbol{2}}}$	LR	0.578 (±0.29)	0.789 (±0.14)	0.790 (±0.16)	0.608 (±0.26)	0.953 (±0.03)	0.917 (±0.05)	12	8
${{\boldsymbol{X}}}_{{\boldsymbol{T}}{\boldsymbol{1}}{\boldsymbol{T}}{\boldsymbol{2}}}$	LR	**0.681** (±0.28)	**0.841** (±0.14)	**0.834** (±0.16)	**0.685** (±0.27)	**0.964** (±0.03)	**0.937** (±0.04)	29	10
${{\boldsymbol{X}}}_{{\boldsymbol{clin}}}$	LR	0.433 (±0.35)	0.716 (±0.18)	0.679 (±0.16)	0.440 (±0.38)	0.953 (±0.02)	0.914 (±0.04)	3	20
${{\boldsymbol{X}}}_{{\boldsymbol{clinT}}{\boldsymbol{1}}}$	XGB	0.446 (±0.37)	0.723 (±0.18)	0.709 (±0.18)	0.473 (±0.35)	0.949 (±0.03)	0.908 (±0.06)	5	6
${{\boldsymbol{X}}}_{{\boldsymbol{clinT}}{\boldsymbol{2}}}$	LR	0.678 (±0.29)	0.839 (±0.15)	0.794 (±0.15)	0.677 (±0.28)	0.968 (±0.03)	0.942 (±0.05)	24	16
${{\boldsymbol{X}}}_{{\boldsymbol{clinT}}{\boldsymbol{1}}{\boldsymbol{T}}{\boldsymbol{2}}}$	LR	0.450 (±0.30)	0.725 (±0.15)	0.719 (±0.16)	0.482 (±0.27)	0.936 (±0.04)	0.888 (±0.06)	18	16

The average and standard deviation (in parentheses) of each performance metric were computed across 20 models, based on 4 folds in the outer loop and 5 repetitions. Highest performances are given in bold. For each dataset, the table displays the best algorithm and its performance metrics: MCC, scaled MCC, AUC, F1 pos, F1 neg, and accuracy. The last two columns report the number of variables included in the model (# variables) and their minimum selection frequency out of 20 times during the RENT variable selection process (RENT selection frequency).

Models based on the clinical data (${X}_{{clin}}$) alone were also assessed (Fig. 1b), but achieved lower performance (MCC = 0.433 for LR). A small increase in the model performance was obtained when the clinical dataset was combined with the pre-therapeutic immune status (${X}_{{clinT}1}$). XGB performed best with an MCC = 0.446. The combination of the clinical dataset with the post-therapeutic immune status (${X}_{{clinT}2}$) resulted in a strong increase of performance (MCC = 0.678). Finally, the combination of the clinical data with the immunological data of T1 and T2 (${X}_{{clinT}1T2}$) resulted in lower performance (MCC = 0.450).

When comparing the predictive performance of models trained on different combinations of the datasets, it is apparent that the model based on immunological data of both the pre- and post-therapeutic immune status (T1 & T2) achieved the highest test performance in the nested cross-validation. Further, the inclusion of both the pre- and post-therapeutic immune status data was necessary to build the highest-performing model. Models trained on the pre- or post-therapeutic immune status alone showed weaker performance. The addition of clinical data did not further increase the model performance. However, the model built on clinical data and the post-therapeutic immune status alone nearly reached the same model performance as the model based on the pre- and post-therapeutic immune status (MCC = 0.681 vs. MCC = 0.678).

### The predictive immune signature consisted of several innate and adaptive cell types

**Fig. 2:**
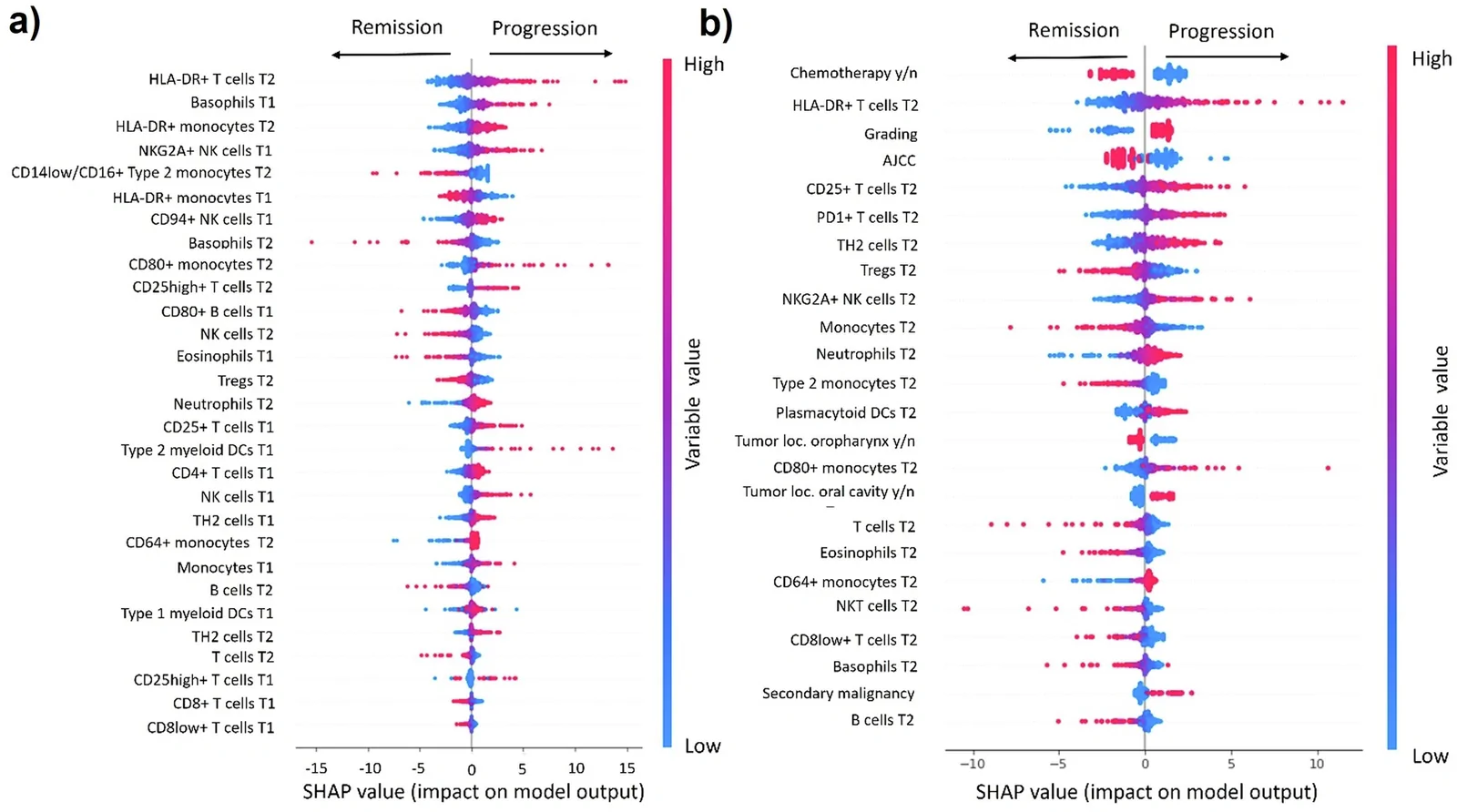
SHAP plots summarizing the predictive parameters for disease-free survival (DFS) in the DIREKHT patient cohort. The figure shows Shapley Additive Explanations (SHAP) summary plots for machine learning models predicting disease-free survival (DFS). DFS = 0 indicates disease-free status, while DFS = 1 indicates disease progression or recurrence. **a** Shows the 29 immunological features selected in the best-performing model (*X*_*T1T2*_). **b** Depicts the clinical and post-therapeutic immunological variables selected in the second highest-performing model (*X*_*clinT2*_). Variables are sorted on the y-axis by importance, with the most important variables shown at the top. The x-axis shows the SHAP value, representing the impact of each variable on the model output. Negative SHAP values indicate a shift towards remission / disease-free status, whereas positive SHAP values indicate a shift towards disease progression. Each colored dot represents one patient. Pink dots indicate high original variable values, and blue dots indicate low original variable values. For binary clinical variables recorded as yes = 1 and no = 0, high values indicate “yes”, while low values indicate “no”. T1 pre-therapeutic time point, T2 post-therapeutic time point, HLA-DR human leukocyte antigen-DR isotype, CD cluster of differentiation, NK natural killer, NKT natural killer T, Tregs regulatory T cells, TH2 T helper 2, DCs dendritic cells, PD-1 programmed cell death protein 1, NKG2A natural killer group 2A, AJCC American Joint Committee on Cancer, y/n yes/no, tumor loc. tumor localization.

The highest-performing model, based on the immunological data of T1 and T2, consisted of 29 predictive immune variables shown in the Beeswarm plot generated with the SHAP package^21^ (Fig. 2a). Out of these variables, 13 originated from the post-therapeutic immune status (T2), while the remaining 16 variables were derived from the pre-therapeutic immune status (T1) of the patients.

The variables are sorted by variable importance in the Beeswarm plot with the least important variables at the bottom and the most important at the top. The five variables with the highest importance for model predictions were primarily innate immune parameters and also HLA-DR + T cells. These T cells had the highest variable importance and high values (red color) for this variable at the post-therapeutic time point pushed the patients further towards disease progression. Also, high values for basophilic granulocytes and NKG2A + NK cells at the pre-therapeutic time point contributed to disease progression. Two post-therapeutic immune status monocyte-related variables (HLA-DR+ monocytes and type 2 monocytes (CD14low/CD16+)) were also important. In contrast to the other four variables, the post-therapeutic type 2 monocytes (CD14low/CD16+) contributed to disease-free survival (remission).

The less important variables consisted of other T cell-related variables, both of the pre- and post-therapeutic immune status. These included the Treg count at T2, the frequency of CD25+ T cells at T1 and at T2, and the frequency of TH2 cells before R(C)T. Many of the T cell-associated variables belonged to the CD4 T cell lineage. The cytotoxic T cells (CD8+), however, were ranked as the least important variables (CD8+ T cells and CD8low T cells at T1). Apart from the T cells, two B cell-related parameters were also important. Further, many innate immune cell variables contributed to the model predictions. More monocyte and NK cell-related variables from both time points, as well as granulocytic cells, were found to be important in addition to basophils, the second most important variable. High counts of pre-therapeutic eosinophils and low counts of post-therapeutic neutrophils were associated with disease-free status. Finally, high pre-therapeutic counts of myeloid-derived dendritic cells were coupled to disease progression.

The second highest-performing model, based on the clinical variables and the post-therapeutic immune status, almost reached comparable MCC as the solely immune-based model. Out of 24 selected variables, 6 originated from the clinical dataset. Clinical factors with highest to lowest relevance were +/−CT, grading, American Joint Committee on Cancer (AJCC) staging, tumor site oropharynx or oral cavity, and the occurrence of a secondary malignancy. The administration of CT pushed the patients towards remission. A low tumor grading was favorable as well, while a higher AJCC staging was associated with disease-free status. Patients with oropharyngeal tumors were predicted to have a better outcome, while oral cavity as a tumor location was associated with disease progression. The immune cell types that were described in detail above for the best-performing signature, were also present in the signature ${X}_{{clinT}1T2}$. The most important immunological variable was HLA-DRlow+ T cells at T2. This variable was also the second most important feature in the signature (Fig. 2b).

To assess the influence of model complexity given the limited number of DFS events (only 8 recurrences in the total cohort of 70 patients), we additionally evaluated the third-best-performing model *X*_*diffT1T2*_ that is based on 12 immune variables only. Although its performance was moderately lower than that of the 29-feature model (MCC = 0.578 vs. MCC = 0.681), it remained meaningful, suggesting that the predictive signal was not solely driven by high-dimensional feature combinations. The retention of several key immune parameters from the other two models further supports the presence of a consistent set of immune cell types that are involved in RT-driven immune modulation. In detail, Tregs, basophils, monocytes, and T cells appeared among the top predictors in all models evaluated, indicating the presence of a biologically relevant pattern. A SHAP plot of this model, as well as a matrix that compares the predictive immune parameters between all three models can be found in Supplementary Fig. 2A, B.

## Discussion

It is currently well-known that R(C)T modulates the local tumor microenvironment (TME) and the systemic immune status of patients^19,20^. However, immunological biomarkers for HNSCC patients receiving R(C)T based on the systemic immune status, e.g. from peripheral blood, are scarce. Thus, within the translational research program of the DIREKHT trial, we aimed to define candidate immune parameters that are associated with DFS in HNSCC patients receiving post-operative R(C)T.

A machine-learning-based workflow was used to identify predictive parameters (variables) for DFS of the patients from immunological and clinical data. Although the number of DFS events was limited, nested cross‑validation provides a robust framework for estimating model performance in such settings and is widely applied in high‑dimensional biomedical studies. In this context, the obtained performance values should be interpreted as reflecting an underlying prognostic immune profile, acknowledging that some variability is expected when event counts are low. These findings therefore highlight biologically meaningful patterns though not yet constituting evidence for clinical deployment.

The models were trained on different combinations of the pre-therapeutic and post-therapeutic immune status of the patients and their respective clinical data. The best-performing model was based solely on pre- and post-therapeutic immunological parameters. The inclusion of clinical parameters did not further improve model performance (Fig. 1a, b and Table 1). However, a model built on clinical parameters and the post-therapeutic immune status reached comparable MCC. It needs to be considered that the clinical variables in this combined model may act as baseline prognostic factors and that this model is intended as a contextual comparison, not evidence of additive predictive utility. In this model, the grading of the tumor, the AJCC staging, the tumor location, the occurrence of a secondary malignancy and whether the patients received CT were among the predictive features, along with numerous immunological features (Fig. 2b). The grading and the staging of the tumor are important predictive factors in the clinics, and thus, it is not surprising that they appear predictive in the model ${{\boldsymbol{X}}}_{{\boldsymbol{clinT}}{\boldsymbol{2}}}$. The impact of the variable tumor location is in line with the previously published clinical results of the DIREKHT trial, where a higher locoregional recurrence rate was found in patients with oral cavity tumors^22^. Recently, an updated combined long-term analysis of the RTOG 9501/EORTC 22931 trials found that adding CT to post-operative RT improved OS in HNSCC patients. This benefit was not limited to extranodal extension or positive resection margins^23^. In accordance, the simultaneous administration of CT was highly predictive of a longer DFS in this analysis of the DIREKHT patient cohort. In the DIREKHT study CT was administered according to current guidelines and only applied in patients with high-risk features. Therefore, the here presented analysis is a strong indication that simultaneous CT improves the survival of HNSCC patients undergoing postoperative RT. The AJCC staging was an important feature for the prediction of the DFS. However, it is puzzling that a higher staging was correlated with higher remission rates in patients. This might be attributed to less advanced oral cavity tumors compared to more advanced oropharyngeal tumors in a non-randomized clinical phase-2 trial, which indicates a confounding factor.

Other clinical variables, such as HPV status, are also well-known predictive factors for the outcome of HNSCC patients^24^. Thus, it was surprising that these clinical parameters did not appear in the highest-performing models. One reason for this finding might be the fact that prognostic clinical parameters such as HPV status, have an impact on the patients’ immune system and might thus be encoded in the patients’ immune status. Indeed, HPV+ tumors are known to have higher T cell infiltration and stronger Interferon γ signatures^25,26^, supporting the theory that HPV status-related information can be found in the peripheral immune status. Consequently, this might be a reason why the best-performing model was built alone on immunological data. The high predictive power of the immune status alone might also be explained by the fact that many of the clinical parameters evaluated here, such as age or gender, are static factors. The immune status however, can reflect the dynamics of the disease progression and the course of treatment, making the immunological parameters more conclusive for the prediction of the outcome of the patients^15,20,27^. In line with that, we found that the integration of the pre- and post-therapeutic immune status has led to the best-performing predictive model in this investigation (Figs. 1 and 2). Since R(C)T can induce immune-stimulating and immune suppressive effects, the dynamics of the immune status during the course of therapy can mirror the individual capacity for immune regeneration or adaptation after R(C)T^20^. In line with that, the prognostic impact of the modulation of post-RT immune parameters such as the neutrophil-to-lymphocyte ratio (NLR) has already been confirmed in different studies on HNSCC^28,29^. Further, the model built on the post-therapeutic immune status alone led to a reasonable model performance (MCC = 0.492), while the pre-therapeutic immune status alone resulted in a poor-performing model (MCC = 0.165) (Fig. 1 & Table 1). This finding further supports the hypothesis that the therapy-induced immune modulation mirrors the individual capacity for immune regeneration and adaptation and is thus of major importance for good prognostic performance.

The immune signature presented here consists of different innate and adaptive immune cell parameters (Fig. 2a). Among the five most important immune parameters were pre-therapeutic HLA-DR+ T cells, basophilic granulocytes and NKG2A + NK cells, and post-therapeutic HLA-DR+ monocytes and Type 2 monocytes. In accordance with HLA-DR+ T cells being most important, further T cell-related parameters were part of the signature, such as pre- and post-therapeutic CD25(high)+ T cells. High values of these parameters were associated with disease progression. Even though HLA-DR and CD25 can generally be considered as activation markers of T cells, the role of these cells in the context of HNSCC is not yet well characterized. In a study on oral SCC, a high HLA-DR+ expression was found especially on PD1-tumor infiltrating lymphocytes (TILs)^30^. In the CheckRad-CD8 (NCT03426657) trial, HLA-DR+ T cells have also been found predictive for HNSCC patients receiving induction chemo-immunotherapy^15^. CD25 is also highly expressed on Treg cells, which are well known to suppress anti-tumor T cell responses^31^. Consistent with that, Tregs were also part of the here presented immune signature.

Further, several monocyte-related variables have been found to be associated with the DFS. HLA-DR expression on monocytes has been characterized as a predictive parameter in different cancer entities and treatment modalities, as well as in HNSCC^4,32,33^. Monocytes expressing no or low-level HLA-DR are generally considered mediators of tumor-induced immune suppression and can be grouped with the myeloid-derived suppressor cells^34^. In the proposed signature, high values for HLA-DR+ monocytes at T1 were favorable, while at T2 these cells were associated with disease progression. This fact further stresses that the dynamics of the immune status need to be considered in predictive models. These dynamics are also depicted in the prospective ImmunBioKHT trial, where immune monitoring revealed that the HLA-DR expression on monocytes was downregulated in HNSCC patients after RT^35^.

Moreover, several NK cell-related variables were present the signature. NK cells are also known to have a prognostic impact in the HNSCC TME^36^. In addition, in peripheral blood, NK cells were found to be predictive as well in an HNSCC cohort undergoing induction chemo-immunotherapy^15^. In the prospective ST-ICI01 (NCT03453892) trial, NKT cells predicted a favorable outcome in metastatic NSCLC and HNSCC patients receiving ICI therapy^4^. Regarding the total NK cell count in our study, high numbers at T1 pushed the patients further towards progression, while high counts at T2 were associated with disease-free status. Once more, this finding stresses the importance of the dynamics of the immune status.

All granulocytic cell types, the basophils, eosinophils and the neutrophils were among the immune cell variables associated with DFS. Nowadays, the NLR is established as a predictive marker in different tumor entities, including HNSCC^29,37^. Similarly, low neutrophil counts were associated with disease-free status in our signature. Further, in a prospective study on metastatic HNSCC patients receiving ICI therapy, low eosinophil counts were associated with a favorable outcome^38^. However, in our signature, high eosinophil counts before R(C)T were favorable for disease-free status. These discrepancies might also be explained by the different disease stages of the patient collectives or the fact that most of the immune monitoring studies discussed here focused on ICI therapy, while our analyses were performed on patients receiving R(C)T. Finally, basophils are a rare cell type in peripheral blood; nonetheless, they were among the most important variables in our signature. Current reports repeatedly highlight basophilic granulocytes as markers for predicting side effects, mostly immune-related adverse events (IrAE)^39,40^. Further, a comprehensive analysis of the immune composition in the TME of HNSCC showed that basophils were enriched in the TME of HPV+ tumors^41^. High counts at T1 were associated with disease progression, while high counts post-RT were favorable. In accordance, the CheckRad-CD8 trial revealed that changes in the basophil count (alongside neutrophils, NK cells, and monocytes) pre- to post-induction chemoimmunotherapy were predictive for a pathological complete response (pCR)^15^. In other tumor entities, such as brain tumors, pre-therapeutically low basophil counts predicted a high risk for human cytomegalovirus-associated encephalopathy during R(C)T^42^.

Given the small number of DFS events in this cohort, the risk of overfitting and model instability is a central limitation of the present analysis, particularly for higher-dimensional models. To explore the stability of the identified immune signatures (see Fig. 2 and Supplementary Fig. 2A, B), we compared the top three models using SHAP‑based feature summaries. The third best-performing model *X*_*diffT1T2*_ is more parsimonious and incorporated only 12 immune parameters, while retaining a substantial proportion of the predictive performance. While this reduced model does not overcome the limitation imposed by the small event number, it suggests that the observed associations are not solely dependent on a large and potentially unstable set of predictors. Across all three models (*X*_*T1T2*_, *X*_*clinT2*_, and *X*_*diffT1T2*_), a consistent subset of immune cell populations contributed most strongly to model output. Specifically, six variables representing four immune cell types (Tregs, basophils, monocytes, and T cells) appeared among the top predictors across the three models (Supplementary Fig. 2B). It is worth mentioning that this convergence emerged across the repeated refitting inherent to nested cross‑validation, even in the presence of relatively few DFS events. The recurrence of the same immune features across independent resampling folds suggests an internally consistent biological mechanism that aligns with known immunological mechanisms in this clinical context. While this consistency does not substitute for external validation, it provides exploratory evidence of a potentially robust underlying immune signature that likely reflects biologically relevant cell types in this therapeutic setting.

In summary, the presented signature, comprising 29 innate and adaptive immune variables from pre- and post-treatment time points, offers valuable insights into the immunological landscape and dynamic changes during R(C)T in patients with oral cavity and oropharyngeal cancer undergoing postoperative R(C)T. Moreover, it once again highlights that classical clinical staging and clinical parameters should be combined with comprehensive biological signatures to improve prognostic assessment. Nonetheless and importantly, the presented signatures should be interpreted as exploratory and hypothesis-generating, and their apparent performance and feature composition require validation in independent cohorts before any conclusions regarding generalizability or clinical utility can be drawn. While the complexity of the signature is currently limiting clinical applicability, it highlights key immune cell types involved in treatment response. In the future, a model that ideally combines key clinical and immunological parameters could be used in clinical routine to identify patients with an increased risk of recurrence. One possible consequence could be that these patients receive more frequent follow-up examinations. Based solely on clinical parameters and PD-L1 expression, it is currently difficult to predict the response to checkpoint inhibitor therapy. Therefore, comprehensive immune signatures in combination with clinical characteristics could help identify patients who would benefit from immunotherapy after R(C)T and furthermore predict, in time, upcoming side-effects. The immune cell types identified across all signatures presented here (Tregs, basophils, monocytes, and T cells) represent promising candidates for mediating RT-induced immune modulation. This exploratory analysis lays the groundwork for future mechanistic studies aimed at elucidating the role of these immune cells during RT in HNSCC. The analysis in this study was based on a limited dataset (*N* = 70), with few recurrence events (*N* = 8), and even nested cross-validation cannot fully mitigate overfitting in such a low-event setting, but the data were generated in a prospective setting. Nevertheless, the apparent stability across the folds does not imply generalizability. Still, this model demonstrates once more the increasing role of artificial intelligence-based methods, such as machine learning, in the field of radiation oncology and a key role of the dynamics of therapy-induced immune alteration for the future definition of predictive signatures. Future validation in larger, independent cohorts will be essential to refine the signature toward a more compact and clinically applicable tool^43,44^. The ongoing DIREKHT02 (NCT06030440) trial, which was initiated in 2024, will provide a crucial dataset for this purpose.

## Methods

### Design of the DIREKHT-01 trial

The DIREKHT-01 trial (registered at ClinicalTrials.gov on 2015-08-19: NCT02528955) is a non-randomized prospective multicenter phase II trial initiated in October 2014 at the Universitätsklinikum Erlangen and completed in October 2021. The study was conducted in accordance with the Declaration of Helsinki and approval was granted by the institutional review board of the Friedrich-Alexander-Universität Erlangen-Nürnberg (No.195_14B). The DIREKHT01 trial was conducted in a multicentric set-up. For all participating centers, approval of the study was granted by the respective local institutional review board (institutional review board of the Universität Regensburg, institutional review board of the Land Berlin, institutional review board of the Landesärztekammer Baden-Württemberg, institutional review board of the Fachbereich Humanmedizin of the Universität Marburg & Universitätsklinikum Gießen & Marburg, institutional review board of the Universität Frankfurt am Main, institutional review board of the Sächsische Landesärztkammer, institutional review board of the Ordensklinikum Linz Krankenhaus der Barmherzigen Schwestern Linz). Informed consent was obtained from all patients. The trial aimed to investigate whether an individual de-escalation of the RT dose and volume in selected patients with HNSCC after surgery was able to reduce late toxicity without impacting the oncological outcome, defined as locoregional control.

The patients selected should either have a low risk for local recurrence or a low risk for contralateral neck metastases. Detailed eligibility criteria are published in Haderlein et al.^22,45^. Based on the individual risk factors, the patients were stratified into three different de-intensification schemes: A) Dose reduction in the primary tumor region to 56 Gy (*n* = 7), B) Omitting the elective contralateral neck (*n* = 95), C) Dose reduction at the primary tumor region and omitting the contralateral elective neck (*n* = 85). The patients received concurrent CT with 5-Fluorouracil (5-FU) and cisplatin according to ARO96-3 trial and, if cisplatin-ineligible, carboplatin with 5-FU. Details on CT administration, patient stratification, target volume definition, dose prescription and RT planning can be found elsewhere^22,45^. Table 2 shows detailed characteristics of the patient cohort.

**Table 2:** Patient characteristics of the DIREKHT01 cohort

Characteristics		Number of patients *n* = 70 (%)
Sex	Male	52 (74.3%)
	Female	18 (25.7%)
Age at diagnosis, years	Median	59.5
Tumor location	Oral cavity	21 (30.0%)
	Oropharynx	48 (68.6%)
	Larynx	1 (1.4%)
AJCC classification (TNM 7th edition)	II	1 (1.4%)
	III	34 (48.6%)
	IVa	35 (50.0%)
pT classification	T1	22 (31.4%)
	T2	34 (48.6%)
	T3	14 (20.0%)
pN classification	N0	8 (11.4%)
	N1	27 (38.6%)
	N2a	14 (20.0%)
	N2b	21(30.0%)
Extranodal extension (ENE)	Yes	16 (22.9%)
	No	54 (77.1%)
Lymphangiosis	L0	61 (87.1%)
	L1	9 (12.9%)
Hemangiosis	V0	67 (95.7%)
	V1	3 (4.3%)
Perineural invasion	Yes	10 (14.3%)
	No	60 (85.7%)
Grading	G1	2 (2.9%)
	G2	21 (30.0%)
	G3	47 (67.1%)
	Unknown	0 (0%)
Midline infiltration	Yes	9 (12.9%)
	No	61 (87.1%)
Resection margin	≥1 mm, <5 mm	33 (47.1%)
	≥5 mm	37 (52.9%)
HPV (only oropharyngeal cancer)	Positive	42 (60.0%)
	Negative	5 (7.1%)
	Not defined	1 (1.4%)
Neck dissection	Only ipsilateral	28 (40.0%)
	Bilateral	42 (60.0)
Concurrent chemotherapy	Yes	30 (42.9%)
	No	40 (57.1%)

The primary endpoint was the locoregional recurrence rate after two years, while secondary endpoints were overall and disease-free survival (OS & DFS) and late toxicity. The results of the above-mentioned clinical endpoints have already been published. The trial met its primary objective. The de-intensification of RT in the pre-defined patient population did not increase the risk of locoregional recurrence, while leading to a favorable toxicity profile^22^.

### Translational immune monitoring program in the DIREKHT-01 trial

To gain insights into the impact of R(C)T on the immune status of the patients and to define predictive immune signatures from peripheral blood, a translational research program was conducted within the DIREKHT-01 trial.

Therefore, longitudinal immunophenotyping was performed before (T1) and after R(C)T (T2), as well as two (T3) and four months after completion of RT (T4) at the follow-up examinations. The immunophenotyping was based on a detailed, flow cytometry-based assay processed directly from whole blood within 24 h after the blood withdrawal. The assay enables discrimination of 34 immune cell subtypes and their respective activation status, finally leading to a total of 45 immunological parameters in total per measurement. The detailed protocol and gating strategy have been published elsewhere^46^. The assay was performed on a Gallios flow cytometer (Beckman Coulter) in the standard filter configuration. For data analysis, the Kaluza Flow Analysis software (Beckman Coulter) was used. Microsoft Excel 2016 was used for further analysis of the flow data, such as the calculation of absolute cell counts.

### Principal component analysis

Principal component analysis (PCA)^47^ is a dimension reduction technique that transforms the information contained in a dataset X, with potentially many variables into a few uncorrelated latent variables. These latent variables, also commonly known as components, are linear combinations of the original variables in X. For each component, PCA provides scores, loadings and the amount of total variance explained by the component. Each row in X, i.e. each patient in this study, has its own component score, which can be visualized in PCA score plots and used in further analysis.

In this study, PCA in combination with one-factor ANOVA (analysis of variance) was used to assess whether there were statistically significant differences between patients in therapy arms B and C based on their immune status. This was done in the following way: (I) apply PCA to the dataset (datasets T1 (${X}_{T1}$) and T2 (${X}_{T2}$)) to acquire component scores for each patient in all computed components; (II) for each component a one-factor ANOVA was performed on the scores, with the therapy arm (B and C) as the factor. Based on the resulting *p*-value, the statistical significance of differences between the patients in the two therapy arms was evaluated. The results on the principal component analysis are reported in the Supplementary file.

### Datasets for training of machine learning models

**Fig. 3:**
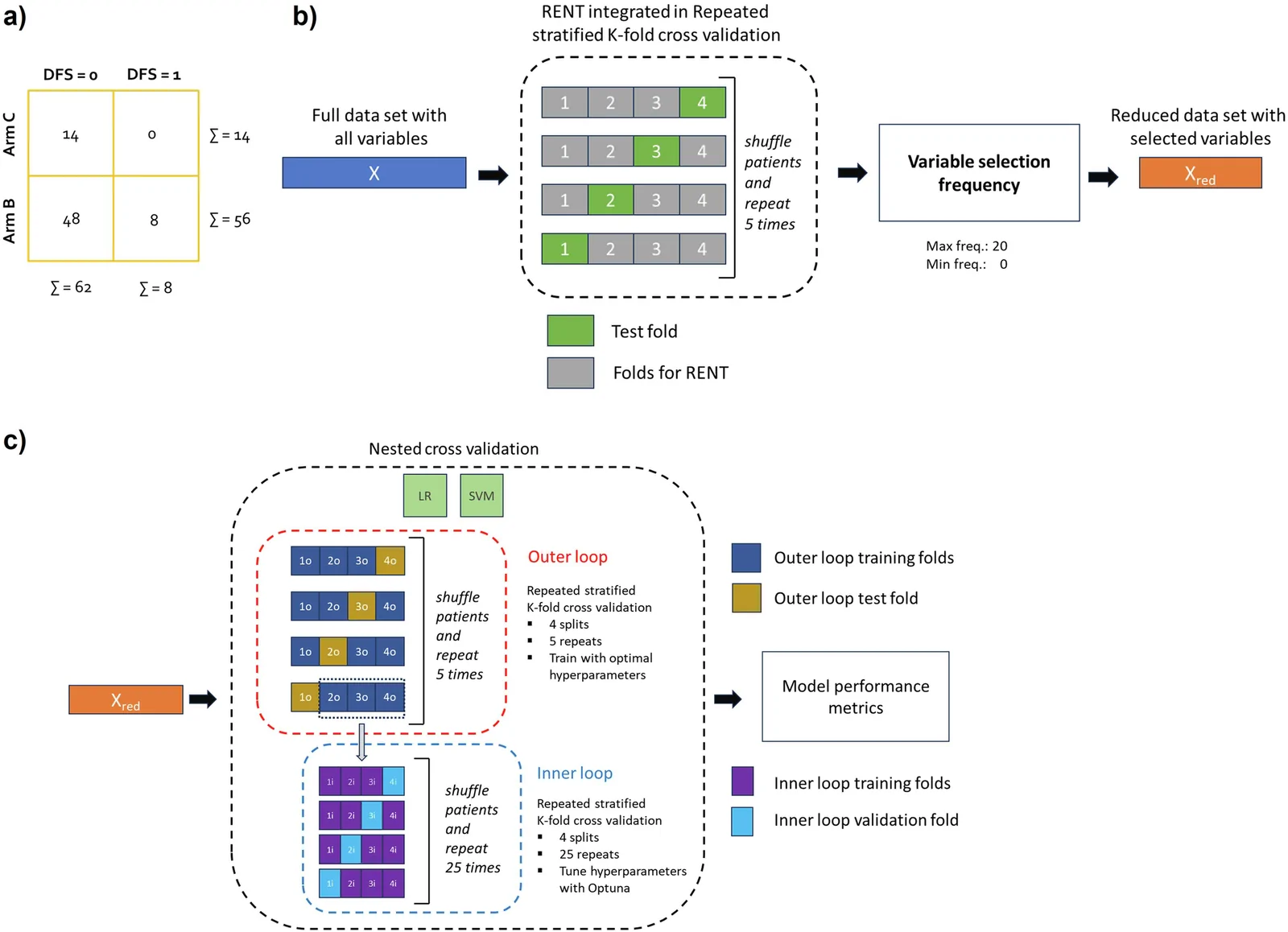
Workflow for training and validating machine learning models to predict disease-free survival (DFS). The data of a cohort of *N* = 70 patients from therapy arms B & C was used in the machine learning (ML) workflow. DFS = 0 indicates no DFS event and DFS = 1 indicates a DFS event. N denotes the number of patients and Σ denotes the total number of patients peer row or column (**a**). The blue box (labeled X) represents the different input dataset combinations that are presented in Table 1. The variable selection using Repeated Elastic Net Technique (RENT) to create a variable selection frequency table is depicted in (**b**). In the RENT procedure, green rectangles indicate test folds, and gray rectangles indicate folds used for training. Patients were shuffled and the procedure was repeated five times. Variables were selected according to their selection frequency, with a maximum possible frequency of 20 and a minimum frequency threshold of 0. The resulting reduced dataset is shown as the orange rectangle labeled X_red_. The reduced dataset X_red_ was used to train different machine learning algorithms (support vector machine (SVM), logistic regression (LR), extreme gradient boosting (XGB)). Model evaluation was performed using nested cross-validation. In the outer cross-validation loop, dark blue rectangles indicate outer-loop training folds, and yellow rectangles indicate outer-loop test folds. In the inner cross-validation loop, purple rectangles indicate inner-loop training folds, while light blue rectangles indicate inner-loop validation folds. The red dashed line indicates the outer loop, and the blue dashed outline depicts the inner loop used for hyperparameter optimization. Model performance was evaluated using different performance metrics (**c**).

Since only 7 patients were recruited in Arm A (dose reduction in the primary tumor region), the analyses were performed on the datasets obtained from patients recruited in Arm B and Arm C. For subsequent analyses, clinical data (patient-/tumor- and therapy-related information like tumor site, HPV-status) as well as longitudinal immunophenotyping data and patient outcome measures (DFS) were used. Due to very few immunological measurements at T3 and T4 (follow-up), only the immunological datasets of T1 and T2 (before and after R(C)T) were considered for the analyses. Both immunological datasets (T1 & T2) were available from 70 patients after the exclusion of patients with missing data. This means that 56 patients of Arm B and 14 patients of Arm C were included in the modeling. DFS was used as the target variable in all machine-learning (ML) models. In Arm B, 48 patients had no disease progression during the follow-up time (DFS = 0), while 8 patients had a disease progression (DFS = 1). In Arm C, all 14 patients remained disease-free during the follow-up (DFS = 0) (Fig. 3a).

Consequently, the input variables for the machine learning workflow were based on three datasets: the immunological dataset at T1 & T2, and the clinical dataset. These three datasets were combined in various ways and used to train machine learning models. Table 3 and Fig. 1a, b show all dataset combinations used in the process.

**Table 3:** Definition and dimension of the datasets used in the machine learning workflow

Dataset number	Dataset name	Number of patients	Number of variables prior to variable selection	Description
1	${X}_{T1}$	70	45	Only variables of dataset T1
2	${X}_{T2}$	70	45	Only variables of dataset T2
3	${X}_{{diffT}1T2}$	70	45	Difference between datasets T2 and T1 (immunological parameter change from T1 to T2)
4	${X}_{T1T2}$	70	90	Variables of T1 and T2 horizontally concatenated
5	${X}_{{clin}}$	70	63	Only clinical variables
6	${X}_{T1{clin}}$	70	108	Variables of T1 and clinical horizontally concatenated
7	${X}_{T2{clin}}$	70	108	Variables of T2 and clinical horizontally concatenated
8	${X}_{T1T2{clin}}$	70	153	Variables of T1, T2 and clinical horizontally concatenated

### Machine learning pipeline

The ML workflow (Fig. 3b, c) was designed to predict the target variable DFS (DFS = 1 representing recurrence; DFS = 0 representing disease-free survival) from several input variables, and, as such is defined as a binary classification problem. The aim was to acquire a model that predicts DFS and also identifies important variables in the model and subsequently links that information to underlying biological processes.

As illustrated in Fig. 3b, c, the machine learning pipeline conceptually consisted of the following sequential steps: First, variable selection using Repeated Elastic Net Feature Selection (RENT)^48,49^, then the construction of a variable selection frequency table and finally a nested cross-validation^50,51^ of machine learning models and computation of several classification performance metrics. Each of the datasets described in the section above and shown in Table 3 was submitted to this machine learning pipeline.

In the first step of the ML pipeline, variable selection was applied to the data stored in the full dataset *X* (step 1 in Fig. 3b). This was conducted by integrating RENT into a repeated stratified K-fold cross-validation scheme that utilized 4 folds and was repeated 5 times. For each repetition, patients were randomly assigned to 4 folds (subsets), ensuring the class ratio between DFS = 1 and DFS = 0 was preserved across all folds. RENT was then applied to the patients in three training folds (gray) and model performance with the selected variables was evaluated by predicting the DFS for patients in one test fold (green). Given the 4 folds and 5 repetitions, a total of 20 RENT variable selections were performed.

From these results, a variable selection frequency table was constructed by counting how often each variable was selected across the 20 RENT variable selections (step 2 in Fig. 3b). The selection frequency may be interpreted as a measure of variable importance, which implies that a variable that was selected 20 times should be considered as important, while less important or non-important variables would exhibit lower or zero selection frequency, respectively. Based on this selection frequency, the variables to be included in the machine learning models, i.e., the reduced dataset *X*_*red*_, were determined.

In the last step (Fig. 3c), models were trained with the following machine learning algorithms on the datasets *X*_*red*_ containing only variables selected by RENT: logistic regression (LR)^52^, support vector classifier (SVC)^53^ and extreme gradient boosting (XGB)^54^. All models were evaluated using nested cross-validation^50,51^ to mitigate overfitting and to obtain performance estimates that are unbiased with respect to hyperparameter optimization. Nested cross‑validation is well established for high‑dimensional biomedical datasets, including settings with limited event counts^55,56^ and provides a principled framework for estimating model performance. Nevertheless, performance metrics derived from sparse‑event data may exhibit greater variability, and the present results should therefore be viewed as highlighting potentially informative immune signatures rather than establishing definitive predictive accuracy.

The outer loop of the nested cross-validation was executed as a repeated stratified K-fold cross-validation, utilizing 4 folds and was repeated 5 times. Within the outer loop, models were trained on the training folds (dark blue) using optimal algorithm hyperparameters determined by the inner loop of the nested cross-validation. The final performance was computed using the outer loop test folds (brown). The inner loop also employed repeated stratified K-fold cross-validation (4 folds, 25 repetitions) on the outer loop training folds (dark blue). The Optuna package^57^ was applied to identify the optimal algorithm hyperparameters.

Model performances were evaluated using the following classification metrics: Matthew’s correlation coefficient (MCC range −1 to +1, where +1 indicates perfect classification, 0 indicates no better than random, −1 is total disagreement) and scaled MCC (range 0 to 1, with 1 indicating perfect performance), F1 score for the positive class (F1 pos, i.e. for patients with DFS = 1) and the negative class (F1 neg, patients with DFS = 0), both ranging from 0 to 1 (where 1 represents perfect precision and recall), Area Under the Curve (AUC, range 0 to 1, with 1 indicating perfect discrimination and 0.5 random performance) and accuracy (range 0 to 1, where 1 indicates perfect prediction) to acquire a comprehensive assessment of model performance. Given that the MCC integrates true and false positives and negatives into a single balanced performance measure that remains informative under class imbalance, we primarily refer to MCC values in the following figures and results. By comparison, the AUC, although widely used in clinical research, may provide overly optimistic impressions of performance in highly imbalanced datasets^58,59^. The highest-performing model was analyzed using the Shapley Additive Explanations (SHAP) package^21^ to visualize, to interpret and quantify the contribution of each feature to the predictions made by the machine learning model.

## Supplementary information


Supplementary information
Supplementary information


## Data Availability

The dataset contains pseudonymized clinical and immunological patient-level data, including longitudinal biomarker measurements and clinical outcome information, which may carry a risk of re-identification, particularly given the specific patient cohort and disease context. Data may be made available from the corresponding author upon reasonable request for scientifically justified purposes, subject to approval by the responsible institutional bodies and/or ethics committee and, where applicable, completion of a data sharing agreement.

## Code availability

The underlying code for the generation of the presented models is available in the following repository: https://gitlab.com/olivertomic1/direkht-npj-code.
