# Supplementary material for: Exploratory immunomonitoring during radiochemotherapy in HNSCC and machine-learning reveal immune parameters associated with disease-free survival
Source: NPJ Precis Oncol. 2026 Aug 31;10:337. doi: 10.1038/s41698-026-01658-w (PMC13530257; doi:10.1038/s41698-026-01658-w)
Supplement: Supplementary file 1 [file 41698_2026_1658_MOESM1_ESM.pdf]

Exploratory immune-monitoring and machine-learning reveal immune parameters associated with disease-free survival in postoperative HNSCC radiochemotherapy

Anna-Jasmina Donaubauer[1,2,4,19], Oliver Tomic[3], Lia Mogge[1,2,4], Sarina K. Müller[4,5], Cecilia Marie Futsaether[3], Kristian Hovde Liland[3], Bao Ngoc Huynh[6], Jens von der Grün [7], Panagiotis Balermpas[7], Max Fleischmann[8], Matthias G Hautmann[9], Felix Steger[9], Christopher Bohr[10], Thomas Hehr[11], Carmen Stromberger[12], Volker Budach[12], Markus Schymalla[13], Rita Engenhart-Cabillic[13], Lukas Kocik[14], Hans Geinitz[14,18], Ursula Nestle[15], Gunter Klautke[16], Claudia Scherl[17], Philipp Schubert[2,4], Stefanie Corradini[2,4], Rainer Fietkau[2,4], Udo S. Gaipl[1,2,4], Benjamin Frey[1,2,4,19 *, #], Marlen Haderlein[2,4,19 #]

[1]Translational Radiobiology, Department of Radiation Oncology, Universitätsklinikum Erlangen, Friedrich-Alexander-Universität Erlangen-Nürnberg, Erlangen, Germany
[2]Department of Radiation Oncology, Universitätsklinikum Erlangen, Friedrich-Alexander-Universität Erlangen-Nürnberg, Erlangen, Germany
[3]Norwegian University of Life Sciences, Faculty of Science and Technology, Ås, Norway
[4]Comprehensive Cancer Center Erlangen-EMN, Erlangen, Germany
[5]Department of Otolaryngology - Head & Neck Surgery, Universitätsklinikum Erlangen, Friedrich-Alexander-Universität Erlangen-Nürnberg, Erlangen, Germany
[6]Department of Medical Physics, Oslo University Hospital, Oslo, Norway
[7]Department of Radiation Oncology, Zurich University Hospital, Zurich, Switzerland
[8]Department of Radiotherapy and Radiation Oncology, University Hospital Frankfurt, Goethe-Universität Frankfurt am Main, Frankfurt am Main, Germany
[9]Department of Radiotherapy and Radiation Oncology, University Hospital of Regensburg, Regensburg, Germany
[10]Department of Otorhinolaryngology, University Hospital Regensburg, Regensburg, Germany
[11]Department of Radiotherapy and Radiation Oncology, Marienhospital, Stuttgart, Germany
[12]Department of Radiotherapy and Radiation Oncology, Charité - Universitätsmedizin Berlin, corporate member of Freie Universität Berlin and Humboldt-Universität zu Berlin, Berlin, Germany
[13]Department of Radiotherapy and Radiation Oncology, University Hospital of Marburg, Marburg, Germany.
[14]Department of Radiation Oncology and Radiotherapy, Ordensklinikum Linz Barmherzige Schwestern, Linz, Austria.
[15]Department of Radiotherapy and Radiation Oncology, Kliniken Maria Hilf, Moenchengladbach, Germany.
[16]Department of Radiation Oncology, Chemnitz Hospital, Chemnitz, Germany.
[17]Department of Otorhinolaryngology, Head and Neck Surgery, University Hospital Mannheim, Medical Faculty Mannheim, Heidelberg University, Mannheim, Germany
[18]Medical Faculty, Johannes Kepler University, Linz, Austria
[19]Bavarian Cancer Research Center (BZKF), Erlangen

*** Correspondence:**
Benjamin Frey
Benjamin.frey@uk-erlangen.de

# Contributed equally as senior authors

Supplementary Results

Stratification into different de-intensification schemes did not differentially impact the immune status

Before combining the dataset of the patient cohorts of therapy arm B and C for the machine learning modelling, we aimed to rule out that the stratification of the patients into different treatment arms might already lead to systemic differences in the immune status. To investigate this, PCA followed by one-factor ANOVA on the PCA scores was performed on the immunological data set X_{T1} of T1 measured prior to therapy. The first ten principal components, capturing the majority of the variance across the dataset (67.2% of the total variance in X_{T1}), were used for visual inspection to assess potential clusters of patients based on therapy arm. An example of such a visualization is the PCA score plot for components 1 & 2 (Figure 2A), together explaining 25% of the total variance (14.8% and 10.2%, respectively). The PCA scores plot revealed no distinct clustering or separation of the patients according to treatment arm B or C. The patients from both cohorts were evenly distributed across the principal component space, indicating no systematic differences in their baseline immune status. The same also holds for components 3 through 10 (not shown). If there were distinct and clear differences between the patients of the two arms, they should be visible when plotting components 1 & 2, as they explain the largest and second largest variances in X_{T1}. In addition to visual inspection, ANOVA was performed on the scores of each component to test for differences between the treatment arms (Table 2). This analysis was used to statistically assess whether the stratification of the patients led to significant differences in the immune status as captured by the principal components. The ANOVA revealed no significant differences in component 1 ($p>0.235$) or component 2 ($p>0.160$) or any of the remaining components. These results suggest that stratification of the patients into the treatment arms effectively balanced immune-related variables, and thus any subsequent treatment-related effects were unlikely to be confounded by pre-existing immunological differences.

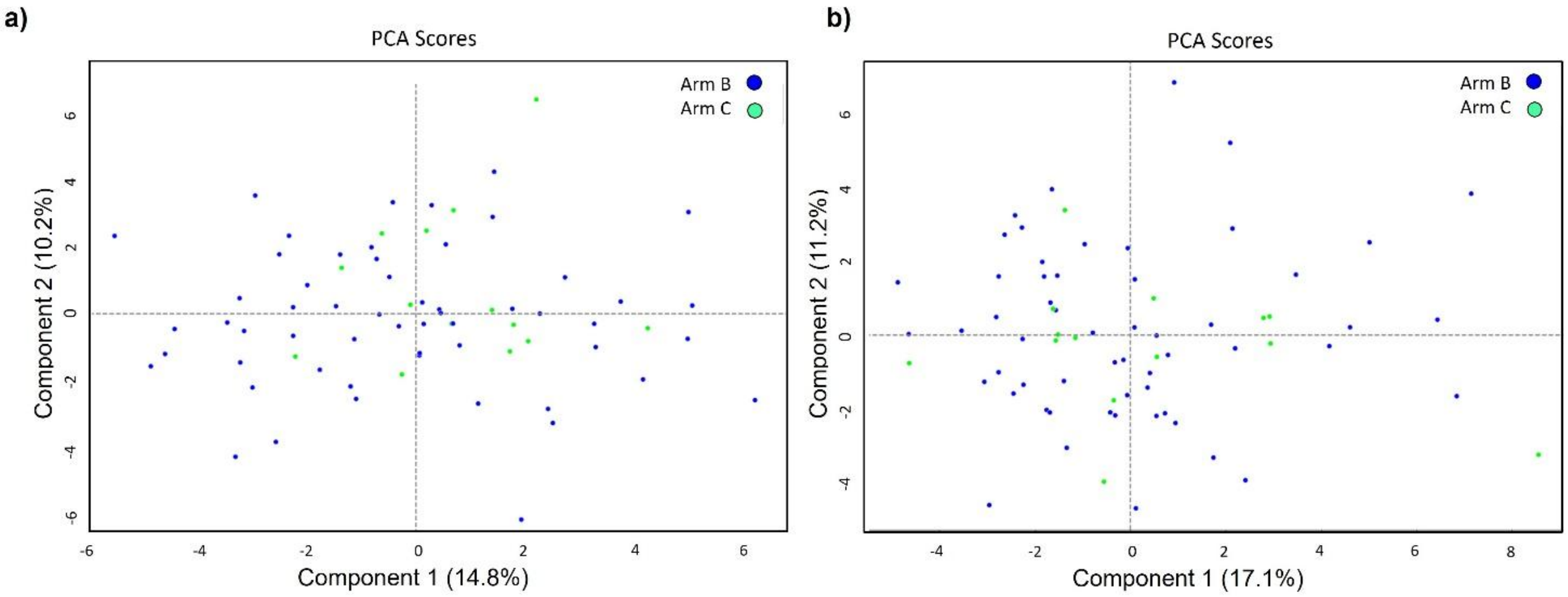

Supplementary Figure 1. Principal component analysis (PCA) to determine the impact of treatment arm stratification on the immune status. Principal component analysis (PCA) was performed to assess whether patients from treatment Arm B and Arm C differed systematically in their immunological profiles. PCA was applied separately to the pre-therapeutic immunological dataset T1 and the post-therapeutic immunological dataset T2(X_T1 and X_T2). Each colored dot represents one patient. Blue dots indicate patients from treatment Arm B, and green dots indicate patients from treatment Arm C. In panel (A), the PCA score plot shows principal component 1 and principal component 2 based on the pre-therapeutic immune status. Principal component 1 explains 14.8% of the total variance, and principal component 2 explains 10.2% of the total variance, together explaining 25.0% of the variance. In panel (B), the PCA score plot shows principal component 1 and principal component 2 based on the post-therapeutic immune status. Principal component 1 explains 17.1% of the total variance, and principal component 2 explains 11.2% of the total variance, together explaining 28.3% of the variance. In both plots, patients from treatment Arm B and treatment Arm C are distributed across the principal component space without clear separation, indicating no systematic treatment-arm-related differences in the pre-therapeutic or post-therapeutic immunological profiles.

Further, we also compared the immunological dataset of the post-therapeutic time point (T2) of the patient arms B and C, to rule out that the different RT de-intensification schemes impacted differentially on the immune status, using the PCA and a subsequent ANOVA analysis as described above. For T2, the first two components explained 28.3% of the variance of the dataset (Figure 2B). Visual inspection of the PCA scores revealed no apparent clustering or separation of the patients according to treatment arm B or C. The patients from both cohorts were evenly distributed across the principal component space, indicating no systematic differences in their post-therapeutic immune status. The same also holds for components 3 through 10 (not shown). The ANOVA revealed no significant differences in component 1 ($p>0.560$) or component 2 ($p>0.549$) (see Table 1). Thus, no systemic immunological differences between treatment arms were found at the baseline immune status and after the different RT treatment regimes.

Supplementary Table 1: ANOVA results of the scores of the principal component analysis for patient stratification into treatment arms. ANOVA was computed for components 1 and 2 for both therapeutic time points T1 and T2.

Cases	Sum of Squares	Df	Mean Square	F	p
***ANOVA – Comp 1* Pre-therapeutic time point (T1)**					
**Therapy arm**	9.539	1	9.539	1.439	0.235
**Residuals**	450.888	68	6.631		
***ANOVA – Comp 2* Pre-therapeutic time point (T1)**					
**Therapy arm**	9.112	1	9.112	2.020	0.160
**Residuals**	306.802	68	4.512		
***ANOVA – Comp 1* Post-therapeutic time point (T2)**					
**Therapy arm**	2.691	1	2.691	0.342	0.560
**Residuals**	534.665	68	7.863		
***ANOVA – Comp 2* Post-therapeutic time point (T2)**					
**Therapy arm**	1.876	1	1.876	0.363	0.549
**Residuals**	351.284	68	5.166		

***Note.* Type III Sum of Squares**

a)

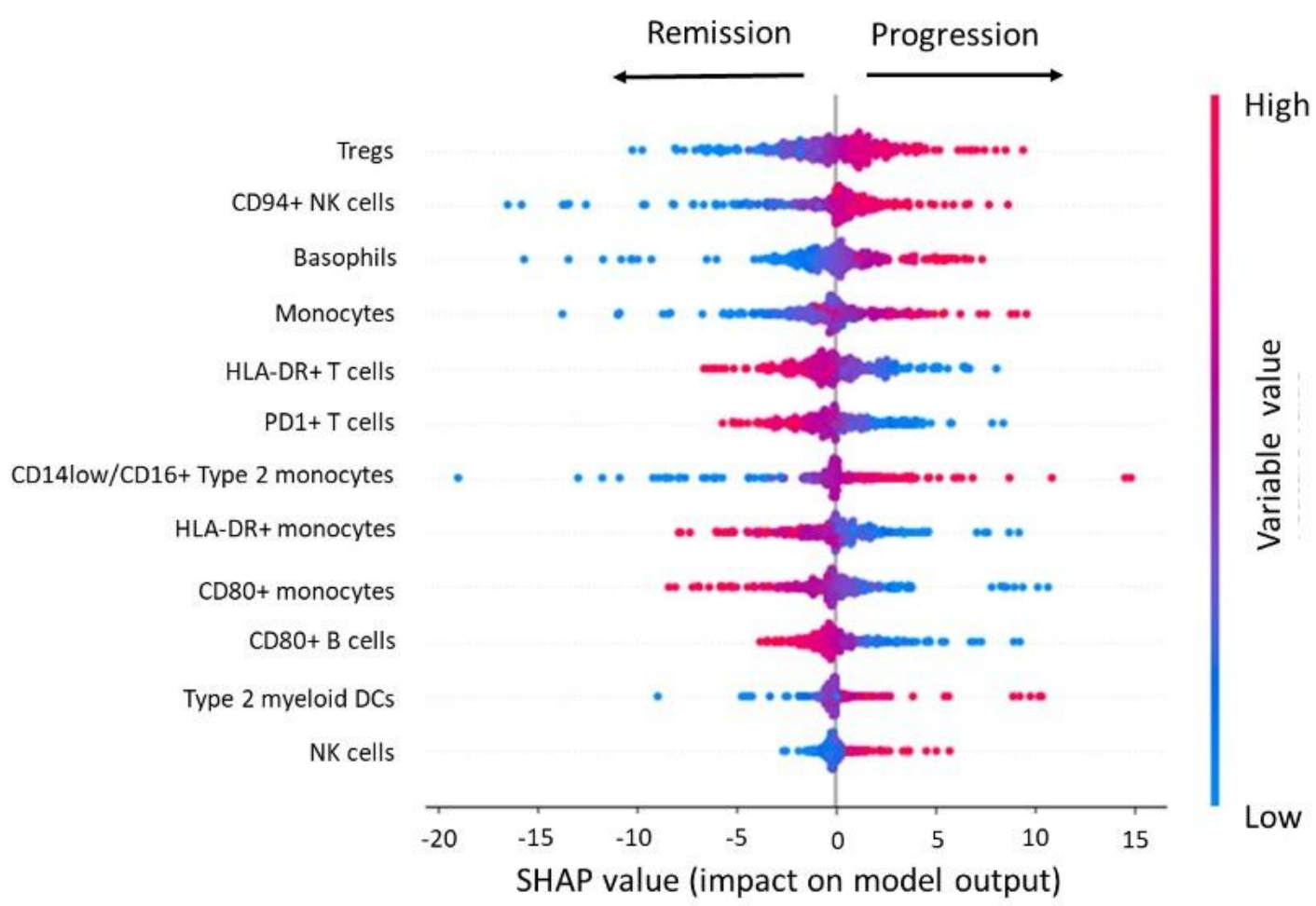

b)

Model (X_{T1T2})	Model (X_{clinT2})	Model ($X_{diffT1T2}$)
HLA-DR+ T cells T2	HLA-DR T cells T2	Tregs
Basophils T1	CD25+ T cells T2	CD94+ NK cells
HLA-DR+ monocytes T2	PD1+ T cells T2	Basophils
NKG2A+ NK cells T1	TH2 cells T2	Monocytes
CD14low/CD16+ monos T2	Tregs T2	HLA-DR+ T cells
HLA-DR+ monocytes T1	NKG2A+ NK cells T2	PD1+ T cells
CD94+ NK cells T1	Monocytes T2	CD14low/CD16+ monos
Basophils T2	Neutrophils T2	HLA-DR+ monocytes
CD80+ monocytes T2	CD14low/CD16+ monos T2	CD80+ monocytes
CD25high+ T cells T2	Plasmacytoid DCs T2	CD80+ B cells
CD80+ B cells T1	CD80+ monocytes T2	Type 2 myeloid DCs
NK cells T2	T cells T2	NK cells
Eosinophils T1	Eosinophils T2	
Tregs T2	CD64+ monocytes T2	
Neutrophils T2	NKT cells T2	
CD25+ T cells T1	CD8low+ T cells T2	
Type 2 myeloid DCs T1	Basophils T2	
CD4+ T cells	B cells T2	
NK cells T1		
TH2 cells T1		
CD64+ monocytes T2		
Monocytes T1		
B cells T2		
Type 1 myeloid DCs T1		
TH2 cells T2		
T cells T2		
CD25high+ T cells T1		
CD8+ T cells T1		
CD8low+ T cells T1		

Supplementary Figure 2. SHAP summary plot for Model ($X_{diffT1T2}$) and comparison of predictive immune populations between the top performing models. Panel (A) shows the Shapley additive explanations (SHAP) summary plot for model $X_{diffT1T2}$, which was trained using the immunological dataset representing differences between the pre-therapeutic time point T1 and the post-therapeutic time point T2. The plot depicts the 12 immune cell variables selected in the predictive immune signature of this model. The x-axis shows the SHAP value, representing the impact of each variable on the model output. Negative SHAP values indicate a shift of the model prediction towards remission or disease-free status, whereas positive SHAP values indicate a shift towards disease progression. The color of each dot represents the original variable value, with pink dots indicating high variable values and blue dots indicating low variable values. Panel (B) compares the occurrence of selected immune cell parameters across the three top-performing models (X_{T1T2}, X_{clinT2}, $Xd_{iffT1T2}$), all of which were evaluated using nested cross-validation. X_{T1T2} denotes the model trained on the combined T1 and T2 immunological dataset, $X_{clinT1T2}$ denotes the model trained on clinical variables combined with immunological variables, and $X_{diffT1T2}$ denotes the model trained on the dataset representing differences between T1 and T2. Yellow cells in the table indicate immune cell parameters that occurred in all three models, green cells indicate immune parameters that occurred in two models and grey cells indicate features that were only selected in one model. This comparison illustrates the degree of consistency across independently resampled models and highlights recurrent immune cell parameters. Abbreviations: T1, pre-therapeutic time point; T2, post-therapeutic time point; DFS, disease-free survival; Tregs, regulatory T cells; NK cells, natural killer cells; NKT cells, natural killer T cells; HLA-DR, human leukocyte antigen-DR isotype; PD-1, programmed cell death protein 1; CD, cluster of differentiation; DCs, dendritic cells; TH2 cells, T helper 2 cells; monos, monocytes.
